# Association of polymorphisms in *LEPR* with type 2 diabetes and related metabolic traits in a Chinese population

**DOI:** 10.1186/s12944-017-0644-x

**Published:** 2018-01-04

**Authors:** Lulu Zhang, Yingfen Qin, Danyan Liang, Li Li, Yaojie Liang, Lulin Chen, Lei Tong, Jia Zhou, Hong Li, Haiying Zhang

**Affiliations:** 1 School of Public Health, Guangxi Medical University, Nanning, 530021 People’s Republic of China; 2 Guangxi Colleges and Universities Key Laboratory of Prevention and Control of Highly Prevalent Diseases, Guangxi Medical University, Nanning, 530021 People’s Republic of China; 3 Department of Endocrine, First Affiliated Hospital of Guangxi Medical University, Nanning, 530021 People’s Republic of China; 4 Third Affiliated Hospital of Guangxi Medical University, Nanning, 530021 People’s Republic of China; 5 Beihai Center for Disease Prevention and Control, Beihai, 536000 People’s Republic of China; 6 School of General Medicine, Guangxi Medical University, Nanning, 530021 People’s Republic of China; 7 Guangxi key laboratory for genomic and personalized medicine, Guangxi collaborative innovation center for genomic and personalized medicine, Guangxi Medical University, Nanning, 530021 People’s Republic of China

**Keywords:** Type 2 diabetes mellitus, *LEPR* gene, Polymorphism, Blood pressure

## Abstract

**Background:**

Leptin acts as a mediator of inflammation and energy homeostasis by activating leptin receptor (*LEPR*). We conducted this study to explore the association of polymorphisms in *LEPR* with type 2 diabetes mellitus (T2DM) and its related metabolic traits.

**Methods:**

We performed a case–control study to investigate the association of polymorphisms in *LEPR* with T2DM and related metabolic traits in a Chinese population, with a total of 922 T2DM patients and 1031 nondiabetic subjects. Polymorphisms were genotyped using MassARRAY assay.

**Results:**

The G allele of rs1327118 was associated with a decreased risk of T2DM in men (*P* = 0.044, odds ratio = 0.707, 95% confidence interval = 0.504–0.991) and the G allele of rs3806318 was associated with increased systolic blood pressure (SBP) in men with T2DM. Besides, the women patients carrying the G allele of rs1327118 showed increased SBP and diastolic blood pressure (DBP) levels, but decreased high density lipoprotein cholesterol (HDL-C) level.

**Conclusion:**

Our results suggest that rs1327118 may be associated with SBP, DBP and HDL-C levels in women with T2DM, and rs3806318 may be associated with T2DM and SBP level in men with T2DM. Further studies with larger sample size or functional experiments focused on exact mechanism are required to verify our observations.

## Background

Diabetes has become a serious public health concern around the world. In 2015, more than 415 million people suffered from diabetes and three quarters of diabetic patients lived in low and middle income countries. At present, China has about 109 million patients with diabetes, and the number of patients is expected to increase to 151 million by 2040, of which type 2 diabetes mellitus (T2DM) accounts for about 95% [1]. T2DM has become one of the major causes of death [2].

T2DM is a multifactorial complex disease that results from the interaction of genetic variants with environmental factors [3, 4], and is characterized by chronic elevated blood glucose levels. A large number of studies [5–10] have shown that genetic factors are considered to be the main cause of individual differences in T2DM and the occurrence of T2DM has obvious genetic heterogeneity. All these studies indicate that genetic variation is an important cause of individual differences in susceptibility to T2DM. There are many genes that interact with environment leading to T2DM. According to the genome-wide association analysis, nearly 60 loci are reported to be associated with T2DM [11, 12]. However, the exact pathogenesis of T2DM is still unclear.

Leptin, first discovered by the group led by Jeffrey Friedman in 1994 [13], is mainly secreted by adipose tissue and is a forerunner of adipokines. Once secreted into the circulation, leptin reaches the central and peripheral nervous systems and then acts by activating leptin receptor (*LEPR*) in the hypothalamus to alter the expression of several neuropeptides, which can regulate food intake and appetite, basal metabolism, reproductive function, bone mass and insulin secretion [14, 15]. *LEPR* is located in chromosome 1p31, and contains 20 exons spanning about 100 kb [16]. *LEPR* can regulate lipid metabolism, blood pressure and blood glucose [16]. The absence of *LEPR* leads to increased food appetite and body fat mass [17]. Additionally, *LEPR* is present in pancreatic β-cells and may be involved in the onset of chronic hyperglycemia and uncontrolled T2DM [18]. Polymorphisms in *LEPR* gene have been investigated in many association studies of obesity, T2DM and diabetes-related complications in recent years [19–23]. These findings suggest that *LEPR* polymorphisms may be associated with the onset and development of obesity and T2DM. Two *LEPR* polymorphisms, rs3806318 and rs1327118, are both located in near gene 5′ of *LEPR*. Several studies have demonstrated that the two polymorphisms in *LEPR* may participate in the progression of cancer and inflammatory response [24–27]. Notably, certain cancer [28–30] and inflammatory response [31–35] may be involved in the pathogenesis of T2DM. And so far limited studies have explored the association between the two polymorphisms and T2DM in different ethnics. Taking this into account, we conducted this study to investigate the association of the selected polymorphisms of *LEPR* with type 2 diabetes in a Chinese population.

## Methods

### Study subjects

We performed a case–control study including 929 unrelated T2DM patients and 1044 nondiabetic healthy subjects from 2010 to 2015 in the First Affiliated Hospital of Guangxi Medical University. All the subjects (≥40 years) who underwent routine physical examination were enrolled. All the participants were requested to undertake a standard 2 h of oral glucose (75 g) tolerance test. T2DM patients were diagnosed according to WHO criteria(a fasting glucose level of ≥7.0 mmol/Land/or a 2 h glucose level of ≥11.1 mmol/L, and/or a self-reported history of T2DM). Those with type 1 diabetes, cancer, or any kind of severe metabolic diseases were excluded. Healthy individuals with normal blood glucose level and glucose tolerance (fasting glucose level of <6.1 mmol/L and 2 h glucose level of <7.8 mmol/L), and no family history of T2DM were recruited as controls. A standard questionnaire was used to assess the socio-demographic information, family history of T2DM and so on. All procedures performed in this study involving human participants were approved by the Ethics Committee of Guangxi Medical University (Nanning, P. R. China). Informed consent was obtained from all subjects.

### Measurements

Anthropometric measurements were conducted by trained medical staff according to standard approaches. Body weight and height were accurately measured to the nearest 0.1 kg and 0.1 cm in light clothing without shoes. Body mass index (BMI) was calculated as weight in kilograms divided by squared height in meters (kg/m2). Blood pressures were measured three times on the right arm using an automated blood pressure monitor (Omron, Kyoto, Japan) in a seated position after an at least 5-min rest, and the mean blood pressure was calculated for further analysis. Biochemical indicators including fasting plasma glucose (FPG), total cholesterol (TC), triglyceride (TG), high density lipoprotein cholesterol (HDL-C), low density lipoprotein cholesterol (LDL-C), hemoglobin A1c (HbA1c), were assessed using a B200 Auto Analyzer.

### Genotyping

Blood samples were drawn with minimal trauma from participants’ antecubital vein in the morning after an overnight fast and stored at −80 °C before analysis. DNA was extracted from peripheral blood leukocytes using the QIAamp DNA blood kit (Tiangen Biotech Co., Ltd., Beijing, China) according to the manufacturer’s instructions. The quality of the extracted DNA was evaluated by electrophoresis on 0.8% agarose gel and the quantity was evaluated by nanodrop device of A260 nm/A280 nm. The MassARRAY Assay Design 3.0 software (Sequenom) was used to design the detection primers and the MassARRAY genotyping system (Sequenom) was used to genotype polymorphisms. Two polymorphisms, rs3806318 and rs1327118 were selected from the International HapMap Project. The genotyping detection rate of the two polymorphisms was up to 95% or over, and finally 922 T2DM patients and 1031 controls were genotyped successfully.

### Statistical analysis

Clinical and laboratory data were compared between groups by unpaired Student’s independent t test or chi-square test. Continuous variables with normal distributions are described as mean ± standard deviation and categorical variables were presented as percentage (%). Chi-squared test was used to compare the allele and genotype frequencies between T2DM group and control group and to test deviations from Hardy–Weinberg equilibrium. We conducted a logistic regression analysis to evaluate the association of investigated polymorphisms with T2DM by odds ratio (ORs) and 95% confidence interval (CI). General and biochemical characteristics were compared between different genotypes using a general linear model (GLM) analysis adjusted for covariates. A level of *P*-value <0.05 was considered as statistically significant, and all statistical tests were two-tailed. Statistical analysis were performed using SPSS Version 17.0 (SPSS Inc., Chicago, IL, USA).

## Results

### Basic characteristics of the study subjects

The basic characteristics of T2DM patients and control subjects are shown in Table 1. The significant differences were found in BMI, systolic blood pressure (SBP), diastolic blood pressure (DBP), FPG, HbA1C, HDL-C, TC and TG between the two groups (*P* < 0.05), and there was no significant difference between the two groups in age, gender and LDL-C level (*P >* 0.05).

**Table 1:** Demographic and biochemical characteristics of the study subjects

	T2DM(*n* = 922)	Non-diabetic(*n* = 1031)	*P*-*value*
Male(n%)^a^	361(39.2)	390(37.8)	0.576
Age(years)^b^	64.9 ± 10.1	64.0 ± 9.5	0.065
BMI(kg/m^2^)^b^	25.1 ± 3.6	24.0 ± 3.2	**0.000**
SBP(mmHg)^b^	139.3 ± 20.6	133.7 ± 19.4	**0.000**
DBP(mmHg)^b^	81.0 ± 11.7	78.9 ± 11.0	**0.000**
FPG(mmol/L)^b^	7.7 ± 2.6	5.5 ± 0.5	**0.000**
HbA1c(%)^b^	6.8 ± 1.6	5.6 ± 0.5	**0.000**
HDL-C(mmol/L)^b^	1.2 ± 0.4	1.3 ± 0.4	**0.000**
LDL-C(mmol/L)^b^	3.1 ± 1.0	3.0 ± 0.9	0.133
TG(mmol/L)^b^	2.0 ± 1.7	1.4 ± 1.0	**0.000**
TC(mmol/L)^b^	5.3 ± 1.5	5.1 ± 1.3	**0.002**

*T2DM* type 2 diabetes mellitus, *BMI* body mass index, *SBP* systolic blood pressure, *DBP* diastolic blood pressure, *FPG* fasting plasma glucose, *HbA1c* Hemoglobin A1c, *HDL-C* HDL cholesterol, *LDL-C* LDL cholesterol, *TG* triglyceride, *TC* total cholesterol

^a^*P*-values were obtained by chi-square tests

^b^*P*-values were obtained by student’s independent t-test

*P* values in boldface are significant

### Genotype and allele distributions

The overall genotypic and allelic frequencies of rs3806318 and rs1327118 in T2DM patients and nondiabetic subjects are shown in Table 2. The genotype and allele distribution of rs3806318 and rs1327118 all conformed to Hardy-Weinberg equilibrium (*P* > 0.05). No significant difference in the genotype and allele frequencies of both polymorphisms was observed between the two groups (*P* > 0.05).

**Table 2:** Distributions of alleles and genotypes between T2DM patients and control subjects

Polymorphism	T2DM	Non-diabetic	Unadjusted *P*^a^	Adjusted OR(95% CI)/*P*^b^
rs3806318	*n* = 925	*n* = 1038		
AA	741(80.1%)	827(79.7%)	0.964	1
AG	173(18.7%)	199(19.2%)		1.038(0.650–1.660)/0.875
GG	11(1.2%)	12(1.1%)		0.971(0.638–1.476)/0.889
A	1655(89.5%)	1162(89.3%)	0.839	1
G	195(10.5%)	128(10.7%)		0.999(0.811–1.231)/0.995
rs1327118	*n* = 922	*n* = 1031		
CC	726(79.7%)	798(77.4%)	0.721	1
CG	178(19.2%)	214(20.8%)		1.063(0.712–1.588)/0.764
GG	18(1.1%)	19(1.8%)		1.011(0.716–1.427)/0.952
C	1630(88.4%)	1810(87.8%)	0.562	1
G	214(11.6%)	252(12.2%)		1.055(0.866–1.286)/0.593

*T2DM* type 2 diabetes mellitus

^a^*P*-values were obtained by chi-square tests

^b^*P*-values were obtained by logistic regression analysis adjusted for age, gender, BMI

*P* values in boldface are significant

The distribution of allele and genotype frequencies of both polymorphisms between male T2DM patients and controls are shown in Table 3. Male individuals carrying G allele of rs1327118 showed a decreased risk of developing T2DM compared with C allele carriers (*P* = 0.044), whereas the CG and GG genotypes did not confer a risk to T2DM. However, no significant difference in the frequencies of both polymorphisms was observed between the two groups in females (Table 4).

**Table 3:** Distributions of alleles and genotypes between T2DM patients and control subjects in males

Polymorphism	T2DM	Non-diabetic	Unadjusted *P*^a^	Adjusted OR(95% CI)/*P*^b^
rs3806318	*n* = 363	*n* = 393		
AA	287(79.1%)	302(76.9%)	0.756	1
AG	73(20.1%)	87(22.1%)		1.020(0.439–2.370)/0.964
GG	3(0.8%)	4(1.0%)		0.880(0.404–1.915)/0.747
A	647(89.1%)	691(87.9%)	0.463	1
G	79(10.9%)	95(12.1%)		0.894(0.639–1.251)/0.514
rs1327118	*n* = 361	*n* = 390		
CC	293(81.2%)	300(76.9%)	0.252	1
CG	65(18.0%)	83(21.3%)		1.491(0.628–3.541)/0.365
GG	3(0.8%)	7(1.8%)		0.512(0.230–1.136)/0.100
C	651(90.2%)	683(87.6%)	0.110	1
G	71(9.8%)	97(12.4%)		**0.707(0.504–0.991)/0.044**

*T2DM* type 2 diabetes mellitus

^a^*P*-values were obtained by chi-square tests

^b^*P*-values were obtained by logistic regression analysis adjusted for age, BMI

*P* values in boldface are significant

**Table 4:** Distributions of alleles and genotypes between T2DM patients and control subjects in females

Polymorphism	T2DM	Non-diabetic	Unadjusted *P*^a^	Adjusted OR(95% CI)/*P*^b^
rs3806318	*n* = 363	*n* = 393		
AA	287(79.1%)	302(76.9%)	0.941	1
AG	73(20.1%)	87(22.1%)		0.990(0.558–1.7561)/0.972
GG	3(0.8%)	4(1.0%)		1.062(0.643–1.753)/0.816
A	647(89.1%)	691(87.9%)	0.746	1
G	79(10.9%)	95(12.1%)		1.053(0.805–1.378)/0.704
rs1327118	*n* = 361	*n* = 390		
CC	293(81.2%)	300(76.9%)	0.644	1
CG	65(18.0%)	83(21.3%)		0.849(0.522–1.381)/0.509
GG	3(0.8%)	7(1.8%)		1.240(0.824–1.868)/0.303
C	651(90.2%)	683(87.6%)	0.627	1
G	71(9.8%)	97(12.4%)		1.112(0.868–1.425)/0.402

*T2DM* type 2 diabetes mellitus

^a^*P*-values were obtained by chi-square tests

^b^*P*-values were obtained by logistic regression analysis adjusted for age, BMI

*P* values in boldface are significant

### Association between polymorphisms in LEPR and diabetes-related metabolic traits

Table 5 shows the demographic and clinical characteristics of T2DM patients according to the presence of the minor alleles of *LEPR* polymorphisms. When the entire population was divided into male and female groups, the G allele of rs3806318 was associated with increased SBP level in men with T2DM after adjustment for age and BMI (*P* = 0.033), but no significant associations were observed in women between rs3806318 and metabolic traits. Women with T2DM carrying the G allele of rs1327118 showed increased SBP and DBP levels, but decreased HDL-C level compared with C/C carriers after adjustment for age and BMI (*P* = 0.004, *P* = 0.026 and *P* = 0.033, respectively), but there was no significant difference in metabolic traits among male patients with different genotypes.

**Table 5:** Demographic and biochemical features of T2DM patients according to the minor alleles of *LEPR* rs3806318 and rs1327118 polymorphisms

Polymorphisms	Wild-type homozygous			Heterozygous + Mutated homozygous			Unadjusted *P* m/f; adjusted *P* m/f^*^	Unadjusted *P*; adjusted *P*^△^
	Male	Female	Total	Male	Female	Total		
rs1327118	CC(*n* = 726)			CG + GG(*n* = 196)				
Sex(%)	293(40.3)	433(59.6)	726	68(34.7)	128(65.3)	196	–	0.161;-
Age(years)	63.8 ± 10.1	65.5 ± 10.0	64.8 ± 10.1	64.9 ± 10.6	65.1 ± 9.7	65.0 ± 9.9	0.442/0.719;-	0.781;-
BMI(kg/m^2^)^a^	25.3 ± 3.7	25.2 ± 3.7	25.2 ± 3.7	25.0 ± 3.0	24.6 ± 3.3	24.8 ± 3.2	0.526/0.113;0.557/0.107	0.091;0.099
SBP(mmHg)^b^	138.8 ± 20.4	138.4 ± 19.6	138.6 ± 19.9	139.6 ± 20.0	143.5 ± 20.0	142.2 ± 22.8	0.787/0.014;0.794/**0.004**	**0.030;0.010**
DBP(mmHg)^b^	82.9 ± 12.0	79.4 ± 10.6	80.8 ± 11.3	81.6 ± 13.2	81.8 ± 12.5	81.7 ± 12.7	0.441/0.032;0.500/**0.026**	0.327;0.181
FPG(mmol/L)^b^	7.8 ± 2.6	7.6 ± 2.6	7.6 ± 2.6	8.1 ± 2.6	7.6 ± 2.4	7.7 ± 2.5	0.399/0.934;0.384/0.879	0.613;0.555
HbA1c(%)^b^	6.7 ± 1.6	6.8 ± 1.6	6.7 ± 1.6	7.0 ± 1.6	6.8 ± 1.6	6.8 ± 1.6	0.230/0.845;0.264/0.966	0.570;0.536
HDL-C(mmol/L)^b^	1.1 ± 0.3	1.33 ± 0.4	1.3 ± 0.4	1.1 ± 0.3	1.26 ± 0.3	1.2 ± 0.3	0.692/0.056;0.397/**0.033**	0.141;0.024
LDL-C(mmol/L)^b^	2.9 ± 1.0	3.2 ± 1.0	3.1 ± 1.0	3.0 ± 1.1	3.2 ± 1.0	3.1 ± 1.1	0.586/0.916;0.973/0.886	0.685;0.862
TG(mmol/L)^b^	2.0 ± 1.8	2.0 ± 1.6	2.0 ± 1.7	2.1 ± 1.1	1.9 ± 1.4	2.0 ± 1.7	0.640/0.472;0.498/0.543	0.819;0.908
TC(mmol/L)^b^	4.9 ± 1.3	5.6 ± 1.5	5.3 ± 1.5	5.0 ± 1.4	5.4 ± 1.5	5.3 ± 1.5	0.468/0.422;0.804/0.313	0.971;0.431
rs3806318	AA(*n* = 741)			GA + GG (*n* = 184)				
Sex(%)	287(38.7)	454(61.3)	741	76(41.3)	108(58.7)	184	–	0.555;-
Age(years)	63.8 ± 10.1	65.5 ± 10.1	64.9 ± 10.1	64.6 ± 10.4	65.0 ± 9.5	64.8 ± 9.8	0.568/0.531;-	0.957;-
BMI(kg/m^2^)^a^	25.4 ± 3.6	25.1 ± 3.6	25.2 ± 3.6	24.8 ± 3.5	24.8 ± 3.5	24.8 ± 3.5	0.188/0.419;0.197/0.399	0.147;0.139
SBP(mmHg)^b^	138.3 ± 20.3	139.4 ± 21.0	139.0 ± 20.7	141.3 ± 20.3	140.2 ± 19.7	140.6 ± 19.9	0.255/0.731;0.177/0.583	0.330;0.214
DBP(mmHg)^b^	82.1 ± 12.3	79.8 ± 11.4	80.7 ± 11.8	84.9 ± 11.8	80.4 ± 10.1	82.2 ± 11.0	0.075/0.659;**0.033**/0.580	0.116;0.076
FPG(mmol/L)^b^	7.9 ± 2.7	7.6 ± 2.6	7.7 ± 2.6	7.6 ± 2.5	7.4 ± 2.4	7.5 ± 2.5	0.390/0.562;0.423/0.548	0.336;0.310
HbA1c(%)^b^	6.8 ± 1.6	6.8 ± 1.6	6.8 ± 1.6	6.6 ± 1.5	6.8 ± 1.5	6.7 ± 1.5	0.313/0.818;0.285/0.781	0.647;0.669
HDL-C(mmol/L)^b^	1.1 ± 0.3	1.3 ± 0.4	1.2 ± 0.4	1.2 ± 0.4	1.3 ± 0.4	1.3 ± 0.4	0.103/0.409;0.151/0.493	0.148;0.153
LDL-C(mmol/L)^b^	2.9 ± 1.1	3.2 ± 1.0	3.1 ± 1.1	3.0 ± 0.9	3.3 ± 1.0	3.2 ± 1.0	0.432/0.213;0.413/0.178	0.175;0.116
TG(mmol/L)^b^	2.0 ± 1.9	1.9 ± 1.5	2.0 ± 1.7	1.9 ± 1.5	2.1 ± 1.5	2.0 ± 1.5	0.598/0.351;0.680/0.353	0.773;0.807
TC(mmol/L)^b^	4.9 ± 1.3	5.5 ± 1.5	5.2 ± 1.5	5.0 ± 1.3	5.7 ± 1.5	5.4 ± 1.4	0.368/0.117;0.396/0.067	0.107;0.053

*BMI* body mass index, *SBP* systolic blood pressure, *DBP* diastolic blood pressure, *FPG* fasting plasma glucose, *HbA1c* Hemoglobin A1c, *HDL-C* HDL cholesterol, *LDL-C* LDL cholesterol, *TG* triglyceride, *TC* total cholesterol

M, male; f, female; Unadjusted *P* values was obtained by chi-square test or student’s test; Adjusted *P* values was obtained by general linear model analysis after adjusting for covariates. However, the gender covariate was not included in after stratification by gender

^a^adjusted for age and gender

^b^adjusted for age, gender and BMI

^*^Results after stratification by gender

^△^Results for the total sample

*P* values in boldface are significant

## Discussion

In this study, we investigated the association of *LEPR* polymorphisms rs3806318 and rs1327118 with T2DM and T2DM-related quantitative traits in a Chinese population. The association of the two polymorphisms in *LEPR* with T2DM susceptibility wasn’t observed in the total subjects, while G allele of rs1327118 might decrease the risk of T2DM adjusted for covariates in men. As to metabolic traits, women with T2DM carrying the G allele of rs1327118 showed increased SBP and DBP levels, but decreased HDL-cholesterol level compared with C/C carriers after adjustment for age and BMI. Moreover, the G allele of rs3806318 was associated with increased SBP in men with T2DM adjusted for age and BMI.

Leptin, as an adipocyte-derived protein, plays an important role in the regulation of appetite and food intake, bone mass, basal metabolism and insulin secretion via *LEPR* [14, 15]. In addition, leptin can stimulate inflammatory response and promote the growth of some cancer cells [36–38]. Numerous studies have suggested that T2DM is a chronic inflammatory disease, and inflammatory response plays an important role in the onset and development of T2DM [31–35]. Meanwhile, certain cancers share many risk factors with T2DM, and are closely linked to the development of T2DM [28–30]. All these studies suggest that leptin and *LEPR* may be involved in the pathogenesis of T2DM. Polymorphisms in *LEPR* are reported to participate in the onset of T2DM [20, 22, 23], and rs3806318 and rs1327118 in *LEPR* are also identified to contribute to cancer [24, 25, 27] and inflammatory response [26]. However, to the best of our knowledge, no study has investigated the association of *LEPR* polymorphisms rs3806318 and rs1327118 with T2DM and related metabolic traits in Chinese population. In our study, the G allele of rs1327118 may decrease the risk of T2DM in men, whereas the CG and GG genotypes did not confer a risk to T2DM. The reason why the association wasn’t observed in genotype distribution may be due to the relatively insufficient male sample.

As mentioned above, *LEPR* can participate in glucose and lipid metabolism. In our study, there was no association between the two polymorphisms of *LEPR* and lipid levels in addition to HDL level. However, both polymorphisms showed a significant association with blood pressure. These results indicated that polymorphisms of rs3806318 and rs1327118 may influence blood pressure in T2DM patients. It has been reported that increasing levels of leptin in diet-induced obesity (DIO) mice drives an increase in blood pressure and re-expression of *LEPR* in DIO *LEPR*-deficient mice also lead to an increase in blood pressure, while humans with loss-of-function mutations in leptin and *LEPR* have low blood pressure [39]. Shannon et al. [40] suggest that the *LEPR*^*L985*^ *(l/l)* mice have higher arterial pressure compared with wild-type controls and a significant association has been previously observed between polymorphisms in *LEPR* and blood pressure [41]. Han et al. [42] also demonstrate that certain polymorphisms of leptin are associated with T2DM and SBP. Leptin’s effects on blood pressure are regulated by neuronal circuits in the dorsomedial hypothalamus [39] and leptin can increase sympathetic nerve activity, and eventually contribute to elevated blood pressure [43, 44]. Furthermore, there is evidence that hypertension and T2DM often coexist and are more likely to contribute to cardiovascular disease [45]. The precise mechanism of these associations remains unclear. Hence further researches are necessary to clarify these associations.

Our study reported significant gender-specific associations of polymorphisms in *LEPR* with T2DM and its related metabolic traits. Sex-specific differences have been previously demonstrated in the onset and development of T2DM and related metabolic diseases [46, 47], and sex-specific genetic background may lead to the different effect of genetic variation on body composition [48]. Gender difference for polymorphisms in leptin and *LEPR*, as well as some other genes have been reported in some association studies of T2DM. Brondani et al. [49] indicate that *FNDC5* variant is associated with changes in blood pressure and lipid profile only in women with T2DM. Ogawa et al. [50] suggest that the serum soluble leptin receptor level in men is significantly higher than that in women. On the contrary, Saad et al. reveal that women have a significant higher leptin level than men at the similar age and body fat [51]. Babic et al. [52] report that plasma leptin is associated with pancreatic cancer risk only in men and leptin levels are higher in women, and rs10493380 in *LEPR* is associated with increased pancreatic cancer risk only in women. There are also many experimental animal models to demonstrate sex differences in metabolism and diseases. It is reported that ablation of *LEPR* causes severe growth hormone deficiency and abdominal obesity in male mice [53]. Nuno et al. [54] discover sex-dependent differences of vascular contractile dysfunction in type 2 diabetic mice. Sex differences in qualitative changes and quantitative changes of metabolic diseases in SDT fatty rats are also observed by Ohta et al. [55]. Taken together, our study also suggest that gender-differences exist in polymorphisms of *LEPR* in a Chinese population.

Our study has some limitations. Firstly, we didn’t measure *LEPR* expression levels which may demonstrate these gender-specific associations. Secondly, behaviors of smoking and alcohol consumption were not evaluated in this study, which may have an influence on the incidence of T2DM. Nevertheless, our study firstly investigated the association of rs3806318 and rs1327118 in *LEPR* with T2DM and metabolic traits in a Chinese population. Further studies with larger male samples are required to verify these gender-specific results.

## Conclusions

Our results suggest that the polymorphism rs1327118 may be associated with SBP, DBP and HDL-C levels in women with T2DM, and the G allele of rs3806318 may be associated with higher SBP in men T2DM patients. Further studies with larger male samples are required to verify our gender-specific results.

## Abbreviations

BMI: Body mass index
DBP: Diastolic blood pressure
DIO: Diet-induced obesity
FPG: Fasting plasma glucose
HbA1c: Hemoglobin A1c
HDL-C: High density lipoprotein cholesterol
LDL-C: High density lipoprotein cholesterol
LEPR: Leptin receptor
SBP: Systolic blood pressure
T2DM: Type 2 diabetes mellitus
TC: Total cholesterol
TG: Triglyceride
